# Correction of water column height variation on 2D grid high-resolution seismic data using dGPS based methodology

**DOI:** 10.1038/s41598-020-75740-z

**Published:** 2020-10-30

**Authors:** Ayobami Abegunrin, Daniel A. Hepp, Tobias Mörz

**Affiliations:** 1 MARUM – Center for Marine Environmental Sciences, University of Bremen, Bremen, Germany; 2 Geo-Engineering.org GmbH, Bremen, Germany

**Keywords:** Geology, Geophysics

## Abstract

Variations in the physical properties of water column usually impede exact water column height correction on high-resolution seismic data, especially when the data are collected in shallow marine environments. Changes in water column properties can be attributed to variation in tides and currents, wind-generated swells, long and short amplitude wave-fronts, or variation in salinity and water temperature. Likewise, the proper motion of the vessel complicates the determinability of the water column height. This study provides a less time-consuming and precise differential Global Positioning System based methodology that can be applied to most types of high-resolution seismic data in order to significantly improve the tracking and quality of deduced geological interpretations on smaller depth scales. The methodology was tested on geophysical profiles obtained from the German sector of the North Sea. The focus here was to identify, distinguish and classify various sub-surface sedimentary structures in a stratigraphically highly complex shallow marine environment on decimeter small-scale. After applying the correction to the profiles, the sea floor, in general, occurs 1.1 to 3.4 m (mean of 2.2 m) deeper than the uncorrected profiles and is consistent with the sea floor from published tide corrected bathymetry data. The corrected seismic profiles were used in plotting the depth of the base of Holocene channel structures and to define their gradients. The applied correction methodology was also crucial in glacial and post-glacial valley features distinction, across profile correlation and establishing structural and stratigraphic framework of the study area.

## Introduction

The physical properties of water column such as density, state of occurrence among others can change significantly over a short period of time. This poses a great challenge on seismic data acquired offshore as a series of independent 2D or 3D seismic grid over time. These changes in the physical properties of water column may result in inaccurate interpretation of geologic features in the sub-sea floor particularly in cases where differences in depth on decimeter scale are critical for the relative stratigraphic classification of morphologic sub-surface structures. Changes in water column properties can be attributed to variation in tides and currents, wind-generated swells, long and short amplitude wave-fronts, variation in salinity and water temperature, pitch, tilt and roll of ship and/or combination of these factors. In nature, these factors could result in real or apparent variations in the water column. While the variation in tides and currents can influence the elevation of the sea surface, variation in salinity and water temperature can drastically affect the water sound velocity^1^. The effects of wind-generated swells during bad weather conditions has been stressed as a factor that can cause short wavelength incoherence in seismic data^2,3^. Accounting for the effects of these variations require offset and depth dependent timing corrections^4^.

Real water column variations can be associated with either internal factors such as thermohaline stratification resulting from changes in water salinity and temperature or external factors caused by changes in water column level resulting from tides, shelf high water and surges due to big storms. Apparent water column variations on the other hand are small scale variations caused by external factors which may be associated with data acquisition like tilt, pitch, roll and shoaling of ship or effects of local wind action on the water surface. Detailed classes of the different ocean waves responsible for water column height variations exists in literature^5^. Long-period waves (storm surges, seiches and tsunamis) can have periods from order of minutes (tsunamis) to few days (big storm surges). Tides which are mostly semi-diurnal have a period band between 12 and 24 h, infra-gravity waves driven primarily by swells have a period range from about 20 to 30 s up to a maximum of about 5 min. Wind sea and swell have a period band of 1 to 20 s. However, swell have small height and less intense dissipation when compared with wind sea. Capillary waves which have period of less than 0.1 s are the shortest period and first wave to be noticed when wind blows on the ocean surface^5^ (Fig. 1).

**Figure 1:**
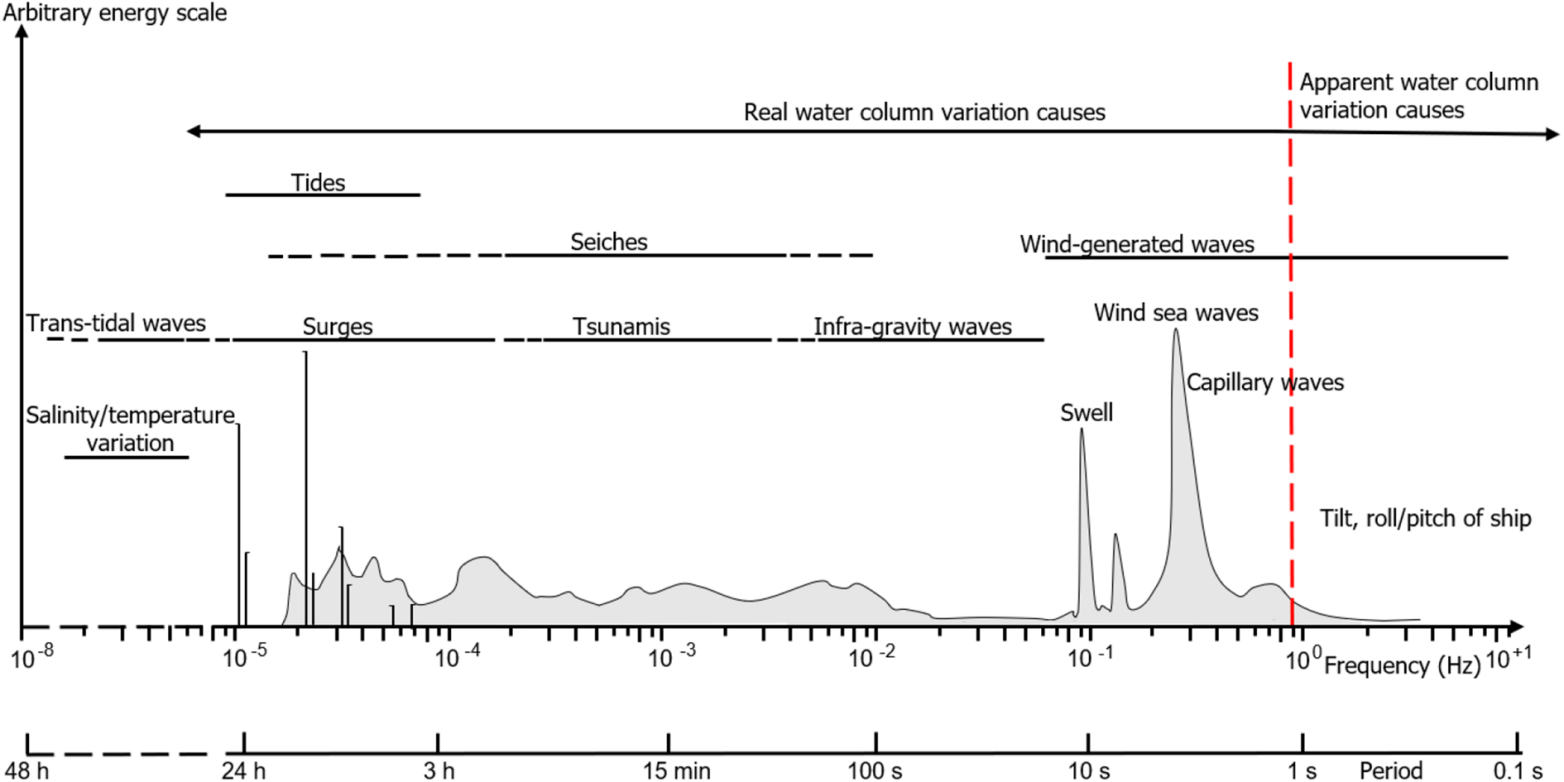
Frequency and period classification of water column height variation causes (Modified after Holthuijsen^29^). The red dash line depicts an arbitrary boundary between the real and apparent water column variation factors.

Being a tidally influenced area, the German North Sea experience a larger tidal range when compared to the German Baltic Sea^6^. Tidal variations can induce lateral discontinuities on reflection seismic gathers^1^ and this in turn hampers the areal-wide interpretation of geophysical profiles or makes it even impossible. A tidal correction, which is as exact as possible, is important for distinguishing various sub-surface morphological structures originating from different processes (e.g. formation of buried tunnel valleys, river valleys, or tidal channels) at different time scales and which on seismic profiles appear to be at similar levels due to erosional processes. These structures can therefore often be distinguished and interpreted only by precise analyses of their altitude in the sediment or their gradients.

In some cases, high-resolution reflection seismic data acquired in shallow water include water column variations in the order of tens of meter relative to the mean sea level. This variation is often ignored by seismic interpreters^7,8^ since a high-resolution deduction of the uppermost meter below the seafloor can be neglected for numerous purposes. Own to complex and high energetic sedimentary processes under glacial, post-glacial and marine conditions, morphological structures originally formed at different times and depositional horizons as well as different sizes and depth scales may now seem to be stratigraphically on the same depth level on seismic profiles. Thus, for example, a determination of the exact depth of valley and channel bases related to a consistent sea level, on decimeter scale, is crucial in distinguishing between different morphological structures, in determining the gradient of rivers or channels and understanding the effect of possible glacial rebound, salt rise and tectonic. This is required for Palaeo landscape reconstruction where it is assumed that the seismic profiles or blocks are acquired using a uniform datum reference.

Correcting for water column variations on seismic profiles requires a good understanding of the origin/cause of the variations in order to account for it in an appropriate procedure. Once obvious acquisition-related factors such as navigation, timing, source and receiver depth errors have been discarded, water-column variations or change in water velocity as a result of change in water salinity and temperature can be identified as the most likely cause of the problem^1^. In shallow water depth areas such as the German North Sea, change in velocity is considered insignificant. This is because there is a whole movement of the water column giving rise to a strong mixing of the water masses and thus preventing any form of thermohaline stratification. In such areas, variations as a result of tides and variations in the sea level due to possible bad weather condition related factors are key contributors to statics observed on seismic profiles. Eliminating the effects of these statics will significantly improve the reflection strength and continuity of seismic data thereby increasing the accuracy of structural and stratigraphic interpretations^7^.

This paper demonstrates the effectiveness of a differential Global Positioning System (dGPS) based methodology in correcting water column height variations using 87 ultra-high-resolution seismic profiles acquired from the shallow German North Sea sector as examples (Fig. 2). The adopted methodology used the mean sea level (MSL) as the datum to which the depth to sea floor was adjusted by adding and/or subtracting a profile length offset obtained from an empirical relation between the orthometric height, vessel height and ship draught.

**Figure 2:**
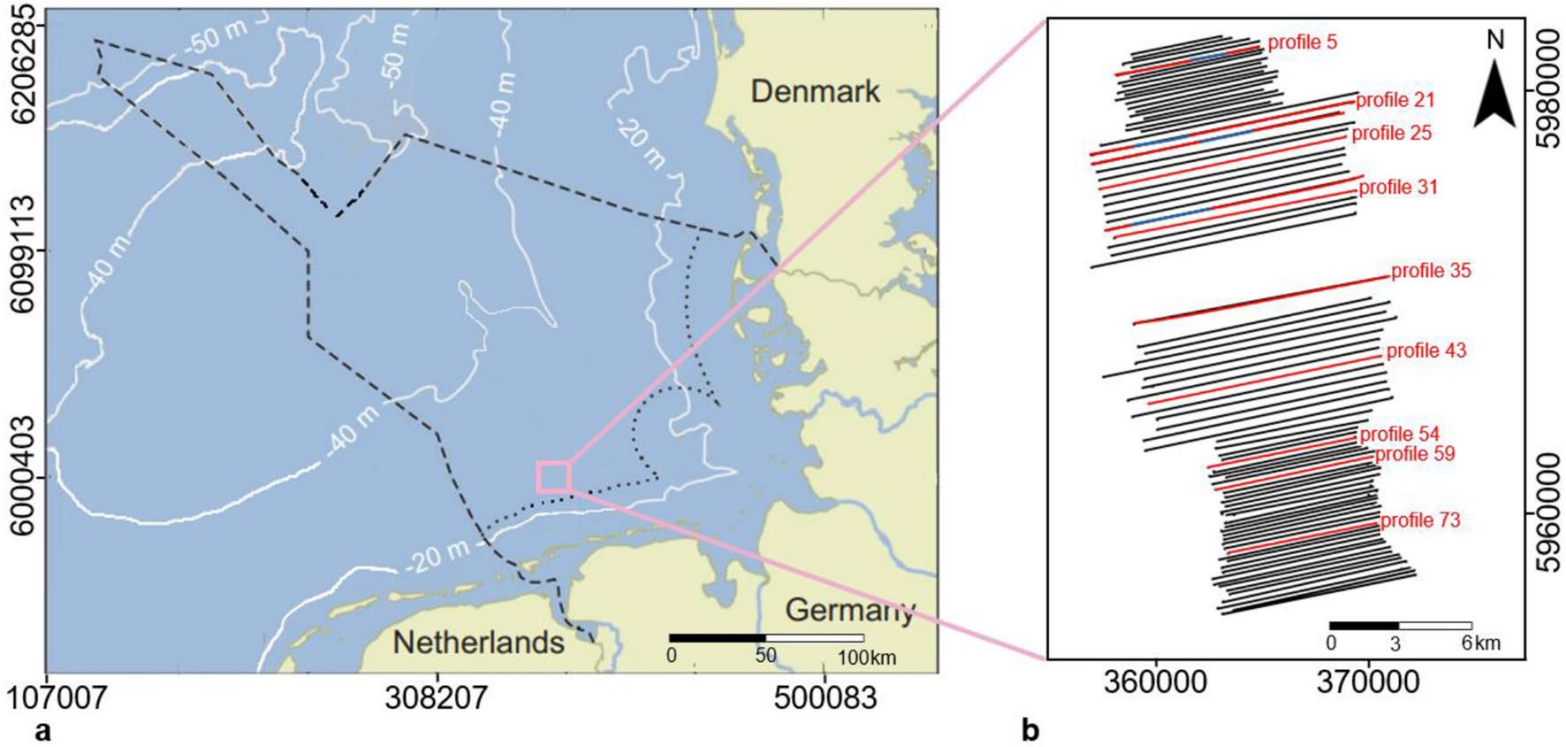
Map of the study area (**a**) north of the East Frisian Islands Norderney and Juist showing the location of the seismic profiles (**b**) acquired during RV Heincke expedition HE499 in 2017. Note: The black dash line is the German Exclusive Economy Zone, black dotted line is the Territorial Waters (12 nautical miles), white solid lines are bathymetry lines and black solid lines are SES geophysical profiles. The key geophysical profiles discussed in this study are the red solid lines while the blue parts represented the sections with prominent geologic features. Figure 2a was modified after Hepp^16^.

### Tidal correction in literature and practice

Tides in most cases are semi-diurnal characterized by two high and two low water levels per day and may cause long wavelength variations. The conventional method for tidal correction is by using predicted tidal tables recorded at nearby tidal gauge stations and this has been utilized in several studies: Balak^7^ for example used tidal charts to compute water column variation in order of 0–5 m relative to the mean sea level for offshore seismic data acquired in the South Heera Field, India. The correction which was about 7 ms significantly improved the data quality and resolution. Despite the applicability of the tidal chart methodology in the entire German Bight area own to the availability of tide gauges and corresponding tidal correction charts^1,6^, the use of this methodology suffers demerits. This method is very time-consuming as several tidal correction charts are required in a larger survey area since each chart applies only to the local area around individual tide gauge. Also, the survey areas are often far from the tidal gauge stations commonly located along the coast and the accuracy, reliability and applicability of the tidal tables are limited and can therefore be questioned^1,6^. Furthermore, in shallow waters, sea storms and wind induced water level variations are not accounted for by tidal charts and their temporal shorter time interval effects can have larger magnitude than tides. In the last couple of decades, recent advances in technology have resulted in non-tidal table-based correction methodology. Xu^9^ employed a non-linear inversion methodology to correct water statics from marine data set obtained from a deep-water environment. The procedure adopted involves automatic picking of relatively statics between sail lines and then eliminating it as a non-linear inversion problem using a priori constraint for final solution uniqueness. Lacombe^1^ proposed a deterministic methodology for resolving water statics as a result of both acoustic velocity and water depth variations and demonstrated its effectiveness on seismic data acquired from the UK continental shelf. Water velocity effects were compensated by direct measurement of root mean square water velocity and arrival time of sea floor reflection at zero offset for each sail line. This was used in computing a spatially constant reference water velocity used in replacing the measured varying real water velocity. On the other hand, changes in water depth was accounted for by averaging dGPS data for the area and computing a mean for the survey from which a differential elevation is computed. Kim^3^ presented a very simple bathymetry-based method for removing tidal effects on high-resolution seismic data acquired from the shallow-water area off the west coast of South Korea assuming a constant seawater velocity. The method used water column height variation computed by finding the difference between depth of the sea floor picked along each sail line and a reference bathymetry. This was then gridded and smoothed by averaging in order to generate the tidal variation for the survey area. The correction along each sail line was extracted from the smoothed grid. Wardell^8^ developed a statistical approach in which tidal corrections are obtained from the redundancy of information contained in the first break in arrival times of the seismic data itself. Common offset spatial averaging was key in computing the correction which was applied to the recorded seismic data.

The general conclusions from the literature overview are that (1) the quality of seismic data acquired in marine environment may be impaired significantly by the state of water column which varies with tide and water sound velocity, (2) while the old method of using tidal correction tables obtained from tidal gauges may still be applicable, time consumption and the distance of the tidal gauge from the survey area in particular poses questions regarding the accuracy and reliability of this method for high-resolution seismic data, and (3) there is a need to develop an alternate tidal correction methodology which is as exact as possible to aid accurate interpretation of sub-surface structures and deducing their approximate depth of occurrence with respect to a fix datum.

### Study area and data base

The study area is located north of the East Frisian Islands Norderney and Juist between northings 5,983,000 and 5,955,000 and eastings 355,000 and 373,500 using the WGS84 UTM Zone 32N Projection (Fig. 2). The study area is characterized by typical shallow sea shelf-sands, sandy or muddy tidal flats or marshes with an average water depth of 25 m dominated by lateral currents. Prominent landforms which constitute the costal landscape are low and small morainic hills flattened by various dunes and gradual movement of wet sediments during the last glaciation^10^. In terms of the geology, different generations of buried valleys spanning in age from Pleistocene to Holocene and each with its own complex origin, development history and infill stratigraphy has been described extensively in literature^11–18^. These valleys are separated by various regional, mostly sandy units of glacio-fluvial or aeolian origin. As oppose to the common NNW–SSE and NNE–SSW preferred axis orientations of the majority of Pleistocene tunnel valleys within the German North Sea, Hepp^15^ identified and described a classical and rare E–W trend for a tunnel valley in the south eastern North Sea. Hepp^16^ also gave a concise seismo-acoustical and sedimentological analyses of a tributary to the Elbe Palaeovalley (EPV) inferred to be the submerged Palaeo extension of the modern Ems River. This Palaeo Ems constitutes to the dewatering system that drained into the EPV within the area^19^. These earlier formed structures are overlain by Modern Mobile North Sea sands of varying thickness forming dunes or layers^18^. Behre^20^ identified various generations of peat formations in Northern Germany associated with regressions which occurred as a result of decrease in the rate of sea level rise during the Holocene. Wetland systems consisting of tidal channels and coastal salt marshes among other structures constitutes other known features within the study area^21,22^. A detailed stratigraphic framework of the Quaternary deposit of the German sector of the North Sea is summarized in Coughlan^17^.

The data used in this study consist of high-resolution seismic and co-recorded differential Global Positioning System (dGPS) data acquired along each of the seismic track lines. These data sets were acquired from the German sector of the North Sea during the RV Heincke expedition HE499 in October, 2017 (Fig. 2). The reflection seismic data were acquired using the ship mounted sediment echosounder Innomar SES2000 medium-100 model with an acoustic power greater than 247 dB (~ 5.5 kW). The SES2000 is a parametric echo sounder system in which two signals of different frequencies are transmitted thereby creating new frequencies when they interact as a result of the non-linearities in the sound propagation. The primary frequency is around 100 kHz and is used for the detection of the sea floor. The secondary frequency, which constitutes the resulting frequency, is much lower than the primary and penetrates deeper into the sea floor. The SES2000 medium-100 model used onboard during HE499 was in multi-frequency mode meaning that secondary frequencies of 4 kHz, 8 kHz and 12 kHz were generated at alternate times. The ping rate was between 0.3 and 0.45 s per frequency and the high primary frequency restricted the aperture angle to 1°. The collected data were converted to SEG-Y-files using the SesConvert tool. A grid comprising a total of 87 seismic profiles with an approximate east–west orientation was acquired in a north–south progression (Fig. 2). North–south profiles as well as sediment cores required for lithostratigraphic studies were not recovered due to extreme bad wave and weather conditions. The seismic profiles of the study area have an approximate spacing of 200 m and cover an area of about 321.60 km^2^. The data were then subjected to the conventional seismic processing procedures. Heave, roll and pitch compensation was carried out onboard by the electronic beam steering depending on the external sensor data of the SES2000 system. The dGPS data were acquired at a sampling rate of 150 m along each sail lines using the Trimble SPS × 61 GPS receiver onboard. The Trimble antenna ellipsoidal height was referenced to the Earth Gravitational Model 1996 (EGM 96). With respect to the dGPS quality, the Trimble antenna was shifted to an improved spot on RV Heincke in February, 2017 prior to the HE499 expedition in October, 2017. Additionally, tide corrected bathymetry data for the entire German sector of the North Sea was sourced from Geopotenzial Deutsche Nordsee (GPDN, https://www.gpdn.de/). The data which was published in 2013 was a compilation over a period of about 10 years. The tidal prediction table obtained for the year 2017 was also sourced from the German Federal Maritime and Hydrographic Agency (BSH). The Norderney gauge station where the tidal values were obtained is situated within close proximity to the study area. Conventional seismic processing and shot to shot high frequency static correction were carried out using the Reflex processing software while the IHS Kingdom Advanced software was used for seismo-stratigraphic interpretation on a standard workstation.

### Methodology for the correction

Tidal correction was carried out on the seismic echo sounder (SES) data using the technique illustrated in Fig. 3 with the mean sea level (MSL) been the reference for all corrections. The basic idea behind this methodology is to use a more precise information obtained exclusively from dGPS measurements in computing offsets from MSL to the water level. The ellipsoidal height (Fig. 3a) along each of the sail lines were obtained from the dGPS data. In most cases in this study, the number of satellites from which these values were derived varies from 7 to 10 indicating a good data quality. The height from the reference ellipsoid to the geoid (Geoidal Height–b) which approximates the MSL was computed using the QuasiGeoid GCG2011 software obtained from the German Federal Office of Cartography and Geodesy. The geoidal height (b) was then subtracted from the ellipsoidal height (a) to obtain the height from the antenna to MSL (Orthometric Height–c). The research vessel has a height (d) of 16.35 m and average draught (e) of 3.93 m. The draught was subtracted from the ship height and the resulting value was deducted from the orthometric heights to obtain the offset from the water surface to the MSL. The offset values calculated along each profile are converted to the unit of time (seconds) by multiplying by a water velocity of 1503 m/s and saved in xyz ASCII format. The water velocity was calculated using Leroy et al*.* 2008 equation^23^ with an average temperature and salinity values of 14.5 °C^24^ and 33‰, g/l^25^ respectively for the month of October, 2017 when the survey was carried out. Bailey^26^ used a simplified Leroy equation (Leroy 1968) to compute mean velocities for the months from May to October when a thermocline develops resulting in a 6 °C or 7 °C temperature difference and obtained a velocity of 1502 m/s. Both velocity values show good consistency over the study area. The offset along each profile was then smoothed using a two-way zero phase filtering interpolation to eliminate outliers. The smoothed profile-length offsets represent the water column height variation for each seismic profile. The final smoothed offset/correction profile was loaded to the trace header of the SES data (f) as a total static for each profile using the Reflex software and the traces were shortened or elongated according to the sign convention of the static correction. By applying this correction to all SES data of the study area now eliminate the effect of tides and any other external influences assuming the MSL as the datum for all the acquired SES data (f′). The adopted workflow is summarized in Eq. (1). The depth of the various geologic structures identified on the corrected seismic profiles were converted from the unit of time (seconds) to meters by multiplying half of the two-way travel time by a propagation speed of 1550 m/s assuming velocity homogeneity within the uppermost sub-sea floor. Since the base of the geologic structures were rarely evenly level, the lowest depth range was selected from each seismic profile. Seismic facies analyses based on reflection strength, continuity and geometry was used in differentiating various lithostragraphical units.

1 $${\text{f}}^{\prime } \, = \,{\text{f}} - \left[ {{\text{c}} - \left( {{\text{d}} - {\text{e}}} \right)} \right]$$

where c = orthometric height, d = vessel height, e = ship draught, f = depth from the transducer to the sea floor, f′ = depth from mean sea level to the sea floor.

**Figure 3:**
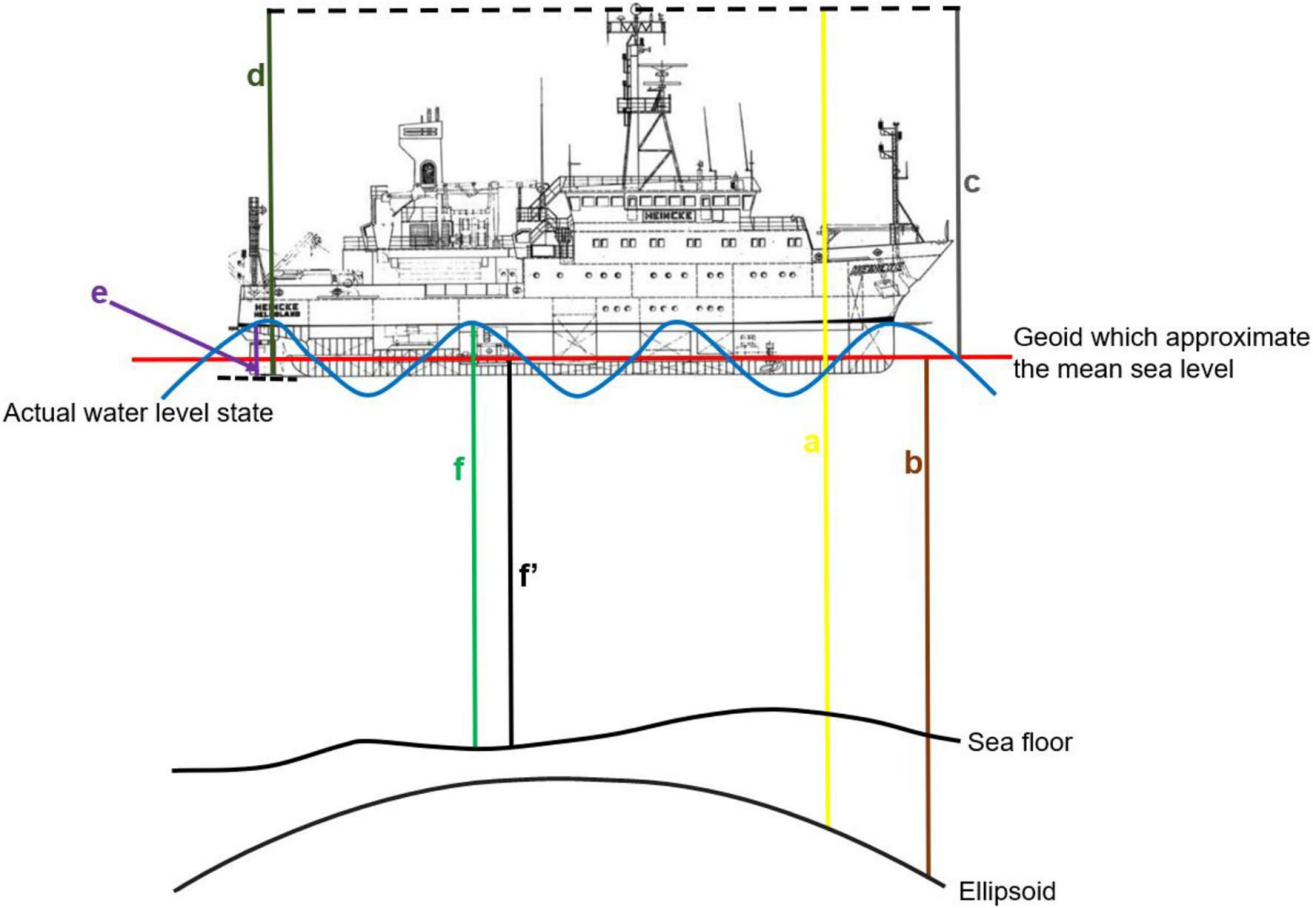
Adopted methodology for water column variation correction to determine the depth from the approximate mean sea level to the sea floor f′; a = height between the ellipsoid and the dGPS antenna (Ellipsoidal Height); b = height between the ellipsoid and the approximate mean sea level (Geoidal Height); c = height between the approximate mean sea level and the dGPS antenna (Orthometric Height); d = ship height; e = ship draught; f = depth from the transducer to the sea floor, draught value was entered to the SES acquisition software; Research vessel image from RV Heincke configuration manual.

## Results

### Water column variation offsets

The offsets of the water level relative to the MSL which constituted the water column height variation for each seismic track line was computed using the procedures outlined above. For each seismic track line and shot, the computed offsets were calculated at an interpolated, equidistance dGPS positions. The estimated low and high water heights and the corresponding water column variation heights along each seismic track line are presented in Table 1. The tidal effects over the course of acquisition of each seismic profile were estimated from the tidal table recorded at the tidal gauge Norderney for the month of October, 2017 when the survey was carried out and also presented in Table 1. At the time of survey, the water level varies from about − 2.3 to 3.9 m relative to the mean sea level. The water column heights range from about 1.1 to 3.4 m with a mean of 2.2 m. From the tidal table, it was observed that the average daily tidal height varies between 4.6 and 5.4 m. However, the contribution of tidal variations to the whole water column height changes over the acquisition period of each profile which is typically between 35 and 90 min varies from about 0.05 to 1.20 m. This constituted in most cases less than 30% of the total variation. Representative examples of the estimated offset types were presented diagrammatically exhibiting three distinct patterns. The first group shows a more gentle variation with offset amplitudes of about 2.0 m along the profile (Fig. 4a,b). The second group showed a sinusoidal offset pattern (Fig. 4c,d) with an average amplitude of 1.5 m while the third group on the other hand were characterized by a marked fluctuation along profile length as shown in Fig. 4e,f. In addition, the plot of the estimated tidal influence revealed a near linear trend over the course of acquisition of each seismic profile (Fig. 4a–f). The approximate water column height variation for the study area as at the time of the survey was obtained by gridding and contouring the computed offsets for each seismic profile (Fig. 5). From the resulting map, it was observed that largest variation occurred near the coast towards the south while smaller variations were characteristic further away from the coast to the north.

**Table 1:** Estimated low water, high water and water column variation heights along each seismic profile.

Profile	Low water height (m)	High water height (m)	Water variation height (m)	Tidal height (m)^a^	Profile	Low water height (m)	High water height (m)	Water variation height (m)	Tidal height (m)^a^
01	− 0.5	1.8	2.3	0.35	45	0.0	2.3	2.3	0.85
02	0.0	2.0	2.0	0.40	46	0.2	2.3	2.1	0.60
03	− 0.1	2.5	2.6	0.40	47	− 1.4	0.8	2.2	0.50
04	0.0	2.0	2.0	0.07	48	0.0	1.8	1.8	0.65
05	− 0.3	2.2	2.5	0.20	49	0.0	2.3	2.3	1.10
06	0.0	1.6	1.6	0.30	50	− 1.0	1.0	2.0	0.40
07	− 1.0	1.9	2.9	0.40	51	− 1.3	0.5	1.8	0.30
08	− 1.5	1.0	2.5	0.40	52	− 2.0	1.0	3.0	0.20
09	− 0.1	2.0	2.1	0.20	53	− 1.6	0.0	1.6	0.08
10	− 1.0	1.3	2.3	0.05	54	− 2.0	0.1	2.1	0.30
11	− 2.0	0.5	2.5	0.10	55	− 0.8	1.0	1.8	0.45
12	− 1.0	1.0	2.0	0.40	56	− 1.0	1.0	2.0	0.65
13	− 0.3	2.0	2.3	0.60	57	0.0	2.2	2.2	0.60
14	− 0.5	1.5	2.0	0.45	58	0.2	2.3	2.1	0.10
15	0.6	2.0	1.4	0.35	59	0.5	2.5	2.0	0.11
16	1.2	2.6	1.4	0.10	60	− 0.8	2.1	2.9	0.20
17	0.6	2.0	1.4	0.10	61	0.0	1.8	1.8	0.20
18	0.8	2.5	1.7	0.20	62	1.5	1.9	3.4	0.30
19	0.3	2.4	2.1	0.40	63	− 1.8	0.1	1.9	0.50
20	0.5	2.4	1.9	0.50	64	− 2.2	1.0	3.2	0.55
21	0.2	1.4	1.2	1.20	65	− 2.3	1.0	3.3	0.40
22	0.8	2.5	1.7	1.00	66	− 2.3	0.9	3.1	0.35
23	1.0	2.5	1.5	0.37	67	− 2.0	0.4	2.4	0.20
24	− 1.4	0.3	1.7	0.39	68	− 2.0	1.1	3.1	0.03
25	0.8	1.9	1.1	0.60	69	− 0.5	2.0	2.5	0.08
26	0.2	1.9	1.7	0.80	70	− 1.0	2.0	3.0	0.30
27	− 0.8	0.4	1.2	0.20	71	0.0	2.0	2.0	0.15
28	0.5	2.1	1.6	0.10	72	− 0.1	2.0	2.1	0.55
29	− 0.1	2.0	2.1	1.00	73	0.5	2.4	1.9	0.45
30	− 0.8	1.2	2.0	0.62	74	− 0.5	2.6	3.1	0.80
31	1.5	3.9	2.4	0.56	75	− 0.5	2.4	2.9	0.05
32	− 0.8	1.8	2.6	0.55	76	− 1.6	1.0	2.6	0.35
33	− 1.0	1.9	2.9	0.52	77	− 1.0	1.0	2.0	0.45
34	− 0.2	2.0	2.2	0.90	78	− 2.0	1.3	3.3	0.65
35	2.0	3.9	1.9	0.80	79	− 2.0	0.5	2.5	0.55
36	− 0.9	0.5	1.4	0.24	80	− 1.5	1.5	3.0	0.40
37	− 0.1	2.5	2.6	0.55	81	− 0.2	1.5	1.7	0.05
38	0.3	1.8	1.5	0.80	82	− 0.3	2.0	2.3	0.55
39	− 0.7	0.9	1.6	0.80	83	0.6	2.8	2.2	0.55
40	1.5	3.4	1.9	0.10	84	− 0.3	3.0	3.3	0.85
41	− 0.7	1.0	1.7	0.20	85	0.3	2.8	2.5	0.35
42	0.2	2	1.8	0.52	86	0.0	2.9	2.9	0.24
43	1.1	3.2	2.1	0.35	87	− 0.5	2.0	2.5	0.29
44	− 1.0	1.0	2.0	0.57					

^a^Estimated tidal values obtained from the tidal table recorded at the tidal gauge Norderney for the month of October, 2017. Tidal prediction does not include the influence of wind and other factors on the sea level (Source: German Federal Maritime and Hydrographic Agency).

**Figure 4:**
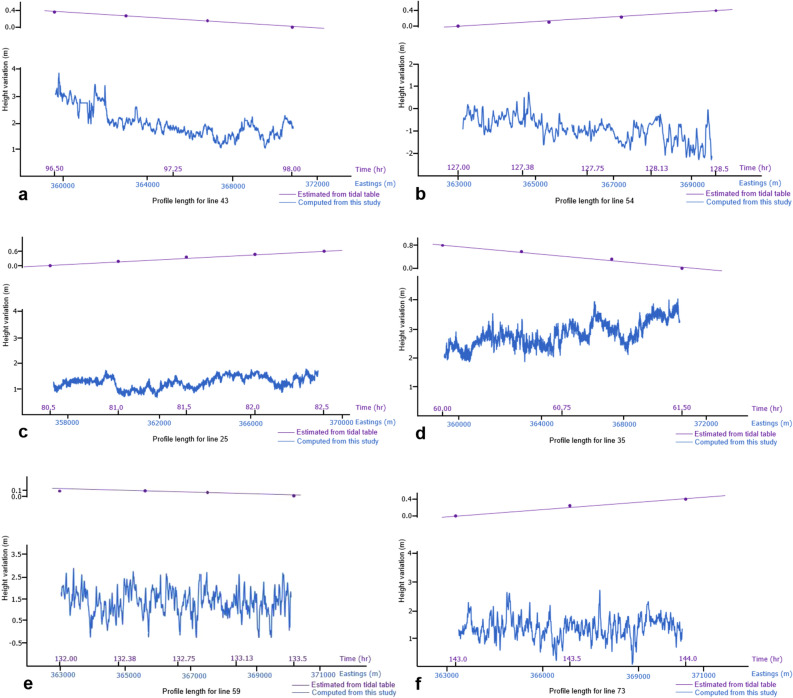
Representative water column variation curves showing a gentle variation (**a** and **b**), sinusoidal pattern (**c** and **d**) and marked fluctuations (**e** and **f**) along profiles (see Fig. 2b for location of the profiles).

**Figure 5:**
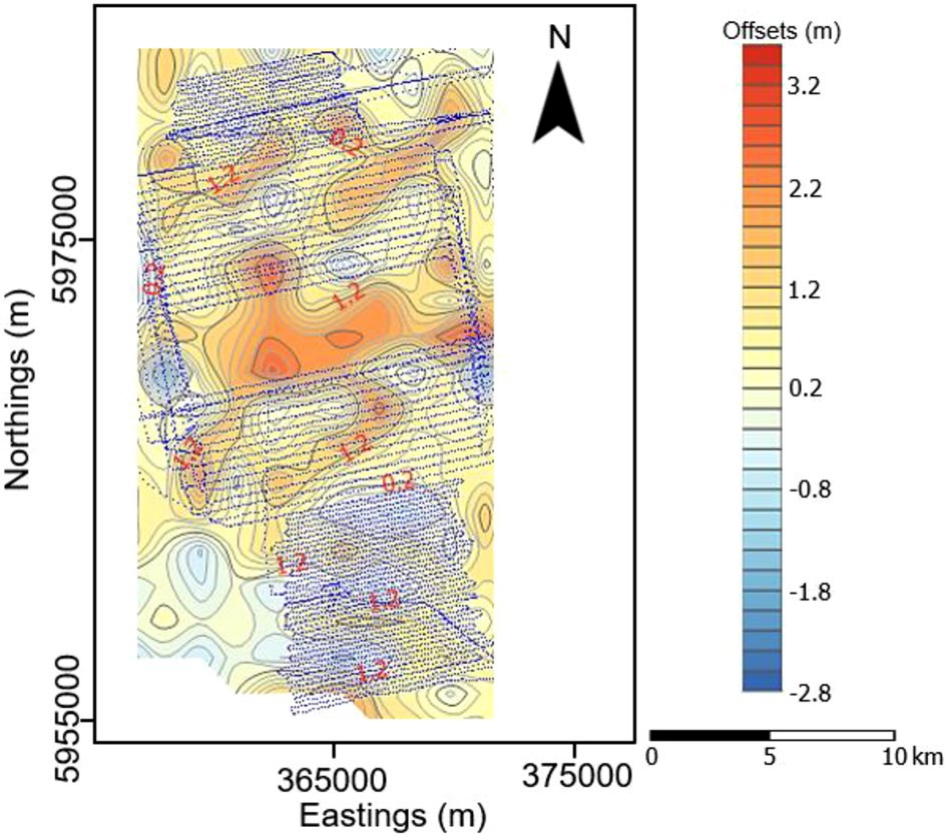
Estimated water column variation map over the study area. The blue lines depict the seismic track lines.

### Application of methodology and comparison of the sea floor from the corrected seismic with existing bathymetry data

Having loaded the corrected seismic profiles into IHS Kingdom Advance software, the sea floor which is typically very distinct on seismic profile was interpreted. Figure 6 shows a SES section for profile 31 after (a) and before (b) the correction for water column height variations and while the structural configuration within the profile was retained, the corrected profile occurs deeper than the uncorrected profile. This was evident as the sea floor picked from the uncorrected seismic profile occur shallower than that from the corrected profile (Fig. 6a). The correction also improved the quality of sub-surface structural deductions from the seismic profile. The sea floor for profile 31 which was extracted from the gridded bathymetry data for the study area was then compared with the picked corrected sea floor. The depth of the sea floor from both the corrected SES and bathymetry profiles show an improved correlation with offset ranging from 0.5 m to a maximum of 2.0 m while the depth of the sea floor from the uncorrected SES profiles occur at a shallower depth with significant offsets (˃ 3.0 m) from the published bathymetry profile (Fig. 7). Furthermore, the depth of the base of a younger channel below the sea floor before and after correction for water column height variation is shown in Fig. 8. It can be observed from Fig. 8 that the depth of the base of the channel plots as scattered points with no definite pattern on the uncorrected geophysical profiles (blue triangles). This implies that an attempt to develop a gradient study on an uncorrected dataset would fail. In contrast, an almost linear depth below MSL trend was observed for the same plot after tidal correction (black circles). This trend of the deepest point of the base of the channel course has a gradient of about 0.00211. Figure 9e reveals the geometry of the younger channel and its stratigraphic relationship with other geologic features (Fig. 9a).

**Figure 6:**
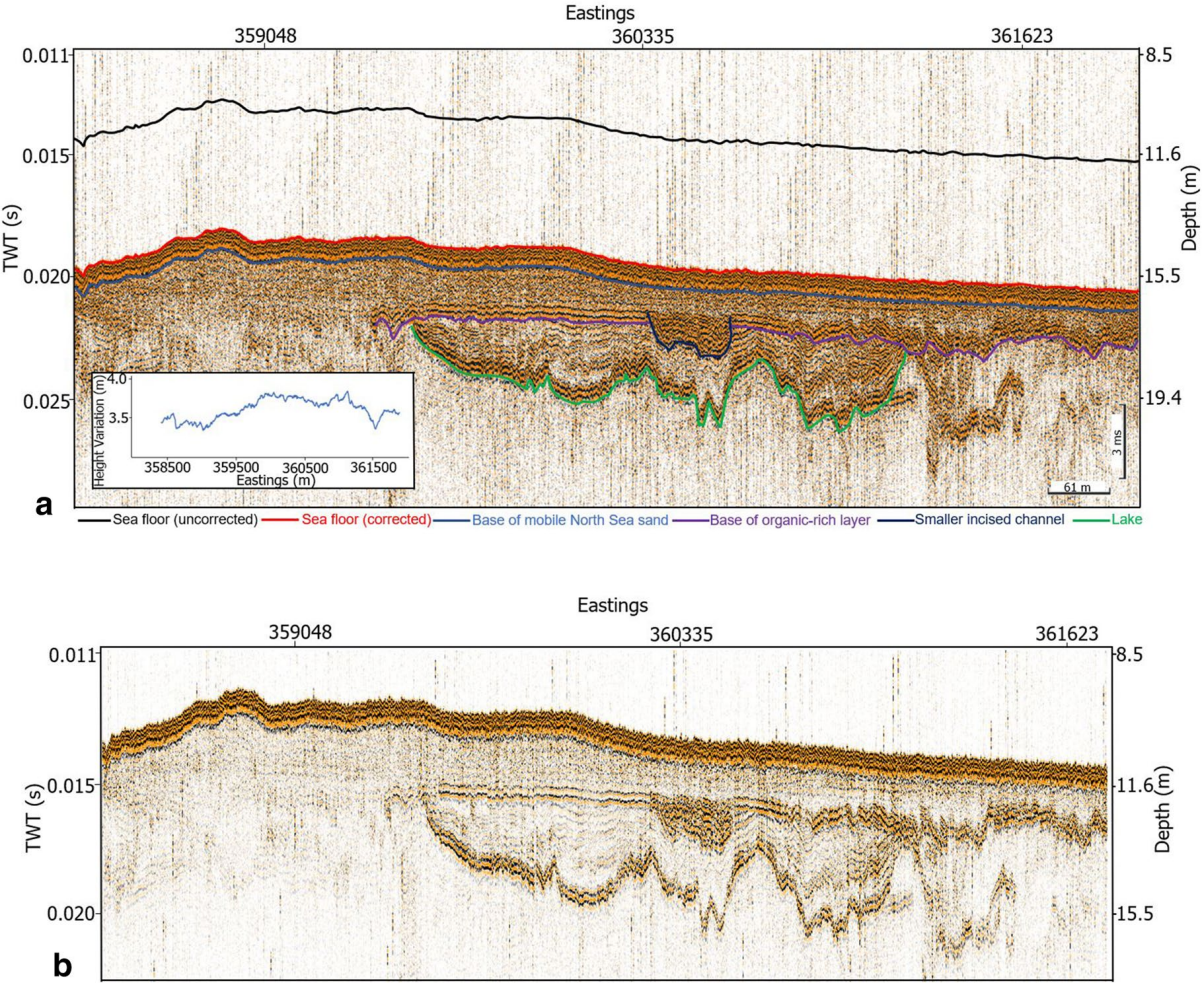
Profile 31 showing the location of the sea floor after (**a**) and before (**b**) tidal correction (see Fig. 2b for location of the profile). Note: The inserted map in (**a**) is the computed water column variation curve for the section shown. Color coding denotes key sub-surface features which are better identified and interpreted on the corrected and processed profiles.

**Figure 7:**
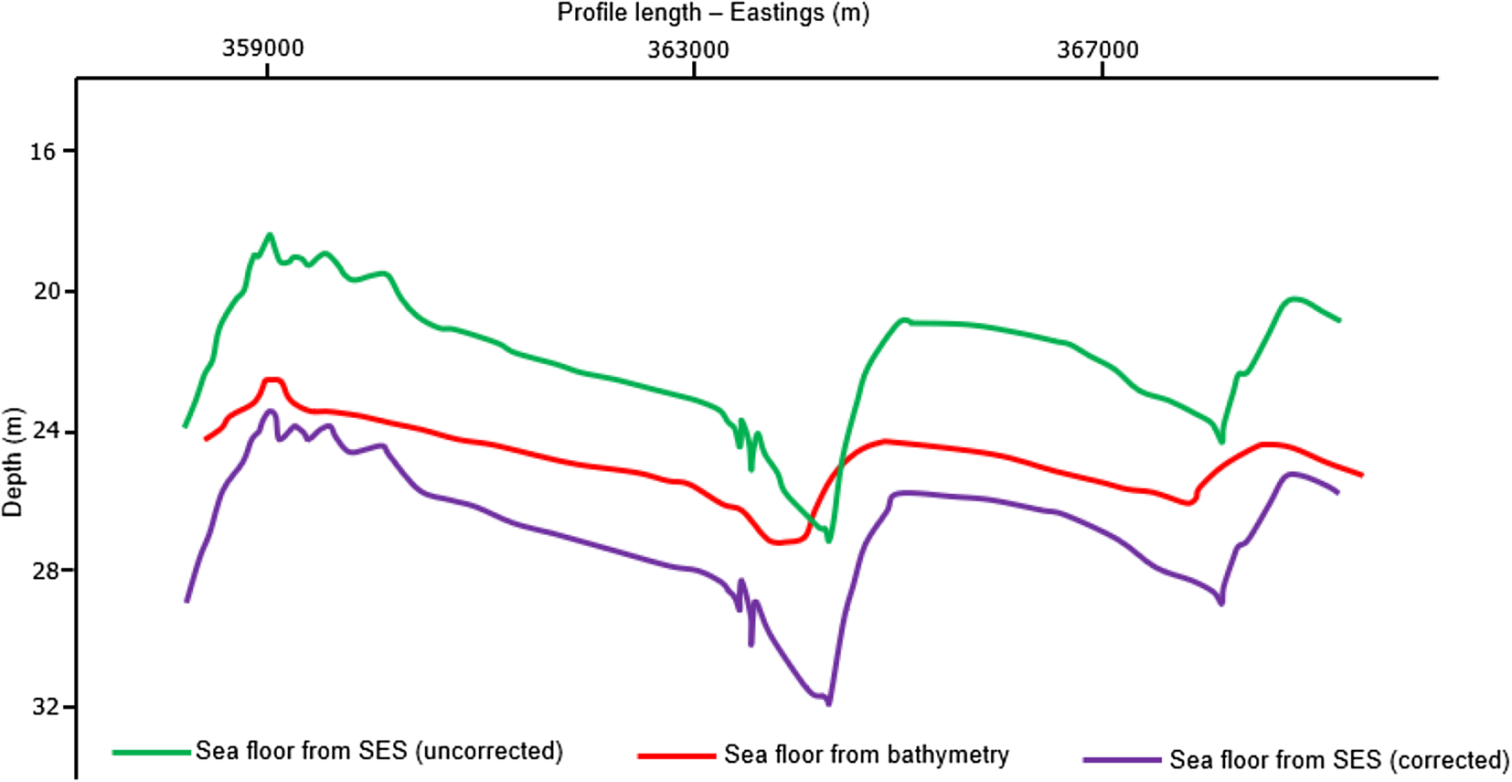
Comparison of the depth of the sea floor from uncorrected SES profile, water column height variation corrected SES profile and bathymetry for Profile 31 (see Fig. 2b for location of the profile).

**Figure 8:**
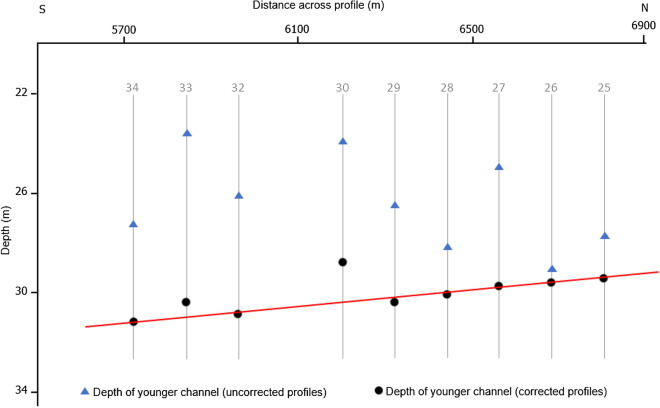
Depth of the younger channel below the sea floor along both the uncorrected (blue triangles) and corrected (black circles) SES profiles (see Fig. 9e for position of the channel base).

**Figure 9:**
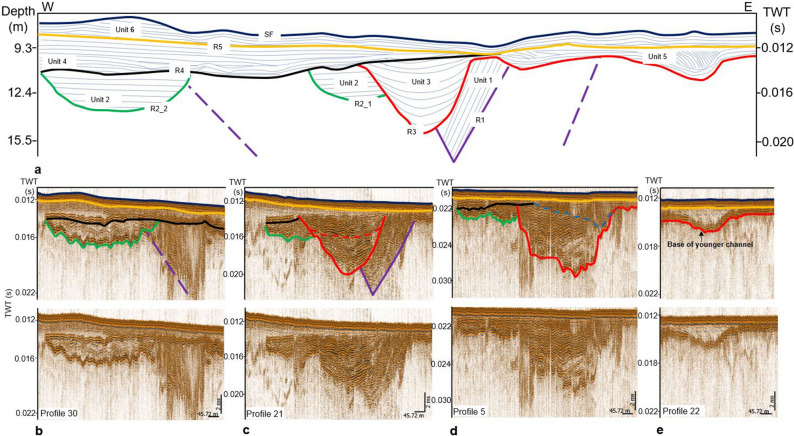
Seismic stratigraphy overview detailing the inter-relationships of different geologic structures over the study area. Major stratigraphic boundaries are highlighted in different colors while the infill sequences are shown in blue. The color lines marked distinct seismic reflectors interpreted as: Modern sea floor surface (blue; SF), base of Holocene sediments (yellow; R5), base of organic rich layer (black; R4), base of Palaeo Ems (red; R3), base of lake (green; R2) and base of the core of Pleistocene channel (purple; R1), (see the blue solid lines in Fig. 2b for location of the seismic sections).

### Major seismic reflectors and units

The schematic composite diagram illustrated in Fig. 9 covering an EW length of about 2.5 km and a resolved depth of approximately 30 m depicts six key seismic reflectors defining major structures and stratigraphic units. The infill seismic expression over the study area was deduced from the corrected profiles as seen in the inset Fig. 9b–e. Some of the major reflectors show former and modern landscape surfaces while other reflectors depict the bases of channels and valleys. Reflector R1 was interpreted as the lower boundary of the core of a tunnel valley capped above by reflector R3. These reflectors marked the lower and upper limit of Unit 1 and it belongs to the oldest geologic structure identified on seismic profiles within the study area. The internal reflector pattern of this unit displays a steeply inclined moderate amplitude, semi continuous to discontinuous internal reflectors (Fig. 9c). Reflector R2_1 was inferred to incised into the tunnel valley and belong to the same generation as Reflector R2_2 based on their approximate erosive depth, architecture and infill pattern. These reflectors defined Unit 2 bounded above by Reflector 4. Unit 2 in the study area appears in two forms expressed on seismic sections as a separate structure and a structure cut by reflector R3 of Unit 3 and it is younger than Unit 1. As evident from the separate structure, Unit 2 displayed a characteristic low to medium amplitude, semi-continuous to continuous internal reflectors unit. However, the base of this unit showed a high amplitude, continuous reflectors (Fig. 9b,c). The upper section of Unit 2 together with Reflector R4 often show a smaller secondary incised channel (Fig. 6). Reflector R3 is a major reflector that incised into the underlying Units 1 and 2 and extends to the eastern part of the study area. Its western extension however has been eroded by the overlying Reflector R4. Both Reflectors R3 and R4 define Unit 3 and this unit is younger than Unit 2 based on cross cutting relationship. The seismic pattern of the infill of this unit revealed a minimum of a two-phase poly infill (Fig. 9c). Seismic facies analysis of the poly infill revealed a strong amplitude and continuous reflector at the base which is overlain by a moderate amplitude and continuous reflector. In the northern part of the study area, Unit 3 show evident of smaller incisions at the upper boundary (Fig. 9d). The strong amplitude and continuous reflectors at the base of this unit extend to the eastern area and forms the base of younger structures (Fig. 9e). Lying above Reflector R4 is a strong amplitude and continuous reflectors defining Unit 4. The area extent of this unit to the east has been eroded by the overlying Reflector R5. The strong amplitude and continuous reflectors from the base of Unit 3 extends to form the base Unit 5. The classic internal pattern is a transparent unit characterized by low amplitude, continuous and dipping internal reflectors (Fig. 9e). In some areas, strong amplitude, continuous and dipping reflectors of this unit was observed to cut the upper area of Unit 3 (Fig. 9d). This indicates a relatively young age relationship for this unit with respect to Unit 3. Unit 6 is bounded below by Reflector R5. Reflector R5 separate recent sediments above from the underlying older sediments across the entire region. Both reflectors R4 and R5 are erosional surfaces of former landscape within the area. Unit 6 is characterized by high amplitude and continuous smaller reflectors on seismic sections (Fig. 9b–e) and bounded above by Reflector SF which is the modern sea floor. None of these structures have an expression on the modern sea floor across the entire area as they were covered by the spatially distributed layer of Unit 6.

## Discussion of results

Differential Global Positioning System information was used to obtain water column height variation using the methodology depicted in Fig. 3. The computed offset values along the seismic profile (Table 1) revealed the genuine existence of water column statics. The computed water column variation ranges from about 1.1 to 3.4 m with a mean of 2.2 m. From the tidal prediction table obtained from the Norderney gauge station for the month of October, 2017, the average daily tidal height varies between 4.0 and 6.0 m for the period of the survey. Over the course of acquisition of each profile (60–90 min), the tidal range varies from about 0.05 to 1.20 m which is less than 30% of the total cause of the variation. This indicates that in shallow water areas where there is no thermohaline stratification, tides contribute only a fraction of the total cause of variations in the water column. The bulk of the variation in such areas can thus be attributed to pitch, tilt and roll of ship and local effects of wind-generated longer wavelength waves and swells which are not accounted for from the tidal gauge predictions.

Diagrammatic representation of the water column variation along each sail line revealed three distinct patterns described as those with gentle, sinusoidal and marked fluctuations patterns (Fig. 4a–e). The tidal variations along each profile behaves close to linearly as shown in Fig. 4a–e for the relatively short time periods of each sail line. This clearly shows the demerit of using predicted tidal tables only for correcting water column height variations for high-resolution seismic studies. This is because tidal tables do not consider the effects of acquisition related factors, influence of wind and waves on the survey vessel itself and other localized factors on the sea surface. In shallow water areas such as the area investigated, these non-tidal related factors can constitute more than 70% of the total statics. This high magnitude effects which cannot be account for based on tidal tables only can greatly degrade the quality of high-resolution seismic data. From the water column variation map generated for the study area for the survey period, high amplitude water column height variations attributed to amplification and shortening in wavelength caused by rapid shoaling toward the coast were observed towards the southern section while smaller amplitude water column height variations were observed in the northern section far away from the coast (Fig. 5). Another factor attributed to the high and low water column variation is the interplay between the water column and sea floor morphology. Towards the coast areas in the south, increased interaction between the sea floor morphology and water column together with the coupling effects of long wavelength waves on the sea bed could result in the observed high amplitude water column variations while the low sea floor morphology-water column interaction could be responsible for the small variations in the northern areas.

The computed corrections along each profile loaded to the SEGY trace header of the SES data as a total static revealed that the corrected profile occurred deeper than the uncorrected profile (Fig. 6). While there is no significant discrepancy in the morphology of the sea floor before and after applying the correction, about 4.0 m difference exist in their depth of occurrence. The sea floor from the corrected profiles which in most cases occur deeper has been referenced to a consistent mean sea level datum. Figure 7 depicts the interpreted sea floor along profile 31 from the uncorrected profile, corrected profile and bathymetry data. It was observed that the sea floor obtained from the bathymetry data was smoother than that of the SES data. Also, on the overall, the sea floor from the corrected profile showed a good consistency with that from bathymetry when compared to the sea floor picked from uncorrected profile. An offset ranging from 0.5 m to less than 2.0 m was obtained from the corrected profiles as opposed 2.0 m to over 3.0 m obtained from the uncorrected profiles. This revealed a better fit between the bathymetry and the corrected SES profiles and clearly indicates the presence of static effects on the seismic profiles during acquisition. The slight discrepancy between the interpreted sea floor from the corrected SES profile and the bathymetry can be attributed to major morphodynamic changes known to characterize the area as the bathymetry data was a compilation over a period of about 10 years and published in 2013. Series of survey carried out over time by BSH within the German North Sea corroborates the existence of intensive morphological changes of the sea floor. In addition, the bathymetry data points have a larger sampling points (data points are about 200 m apart in all directions) leaving out details which were captured in the SES (shot point to shot point distance of about 4 m along profile length). Both Figs. 6 and 7 re-iterated the importance of water column variation correction on seismic data for detailed sub surface interpretation as the SES profiles after applying the correction is not only corrected for static effects but also now referenced to a consistent mean sea level datum.

The stratigraphic position of the base of a younger channel on both uncorrected and corrected profiles was again used to demonstrate the importance of water column variation correction as shown in Fig. 8. Figure 8 depicts the depth of the deepest point of the base of the channel across profiles. From the depth of the base of this channel, it was possible to deduce the trend and gradient of the channel from the corrected profiles which was difficult to decipher from the uncorrected profiles. The channel course has a gradient of about 0.21% and this value together with the width-depth ratio of 2.60, sinuosity of 1.20, entrenchment ratio of 1.38, inferred channel material (sand), cross-section and plan view suggest a river type G5c based on Rosgen^27^ natural river classification (see Fig. 9e for the channel outline). A precise water column height variation methodology as described in this study is critical in shallow and very flat environment with small gradient. This implies that detailed geological interpretation such as gradient studies which can be used, for example, in the identification and classification of river systems and their underlying processes, areal-wide deduction of the approximate stratigraphic positions and trends of sub-surface morphological features will be obstructed on uncorrected geophysical profiles. In areas with complex geologic processes such as erosion and subsequent redeposition, the true stratigraphic position of geological features obtained from corrected seismic profiles is indispensable in distinguishing structures from different time scale (from glacial to post-glacial) and developing a relative stratigraphic model for the area.

Owning to the strong impact of erosion and re-deposition within the study area, glacial and post-glacial structures appeared on first sight indistinguishable as both structures now occur relatively at the same depth level below seafloor on seismic profiles (Fig. 9c, Units 1 and 3). Areal-wide interpretation of these morphological structures on the corrected geophysical profiles helped in distinguishing them based on their approximate true stratigraphic positions. This was difficult to determine on the uncorrected profiles because a structure can occur at different depth positions across profiles from the same area. This was evident from Fig. 8 as the same structure displayed an irregular depth positions across profiles. Also, a discrepancy of about 4 m (about 5.16 ms two-way travel time) existed between the sea floor picked from the uncorrected and corrected seismic profiles (Fig. 6). The implication of this observed discrepancy is that assuming a first-hand velocity homogeneity within the uppermost 30 m below the sea floor, a structure can occur at about ± 4 m relative to its true stratigraphic position within a depth of 30 m. Therefore, distinguishing different sub-surface structures formed at different geological times in a tidally influenced complex shallow water area will be practically difficult if not impossible without a detailed shot to shot water column statics correction.

Seismic interpretation was carried out on both uncorrected and corrected seismic profiles and the relative stratigraphic position of the various identified morphological structures on the corrected profile is shown in Fig. 9. Six major reflectors and their corresponding stratigraphic units were identified in the study area based on seismic facies characteristics. The major reflectors depicted a marked change in impedance contrast as a result of change lithology and reflects the bases of geological structures as well as former and modern landscape surfaces. Based on the major reflectors and units, a local stratigraphy model for the study area was developed (Fig. 9a). Figure 9a also revealed the relative stratigraphic relationship of major identified structures in the study area both in space and time. Due to the lack of sediment cores and other ground truth data, the lithology of each of the identified seismic stratigraphic units were inferred based on previous studies in the area^15–18,28^. The oldest sediment belongs to a large tunnel valley defined by Unit 1. Comparable tunnel valley in the area described by Hepp^15^, Lutz^28^ and Coughlan^17^ suggest that this unit is made up of glacio-fluvial sand deposited in the Pleistocene. This was followed by the infill Unit 2 defined by Reflectors R2_1 and R2_2. The horizontally stratified infill units defined by these reflectors are inferred to be the same based on their approximate erosive depth, architecture and infill pattern. Where the unit is well developed (R2_2), the infill is transparent to semi transparent interpreted to be made up of sandy materials mixed with some fine materials (clay/silt). Unit 3 which incised these earlier formed structures was interpreted to be the Palaeo Ems, the submerged extension of the modern Ems river described by Hepp^16^. The base of this unit which is similar to the base of Unit 2 based on seismic facies characteristics is composed of organic rich layer described as peat by Hepp^16^. Studies by Hepp^16^ revealed that the peat in Unit 3 was overlain by intertidal or marine deposits of clays or silty clays. Careful examination of the internal architectural pattern of this unit revealed a minimum of a two-phase poly infill. Unit 4 then overlies these structures except for where truncated by later formed structures. The characteristics dipping reflectors of Unit 5 were then formed and are inferred to be composed of Holocene sand and/or a mixture of Holocene sand and silt. The whole area was covered by Unit 6 whose base is an erosive Reflector R5 that cut off the area extent of Unit 5 towards the eastern part of the study area. In comparison with the studies by Hepp^16^ and Zeiler^18^, this unit represents the widely distributed recent mobile North Sea sand deposits. This 2–3 m thick sand blankets the older structures within the study area. The sea floor within the study area was depicted as seismic reflectors SF.

## Conclusion

The history of water column statics correction is a research topic with a long tradition which has received special attention in recent decades. Different methods exist for correcting variations in water statics observed on seismic reflection data as a result of changes in water depth due to tides, drastic effect of velocity, acquisition related factors and/or combination of these factors. The data set used for this study was acquired as a series of independent acoustic lines over time with water depth ranging from about 15 to 35 m. Due to the duration of the acquisition, water column height variations was observed within the seismic surveys. Own to the shallow water depth within the study area, change in velocity as a result of change in salinity and temperature was considered insignificant. This was because there was a whole movement of the water column giving rise to a strong mixing of the water masses and thus preventing any form of thermohaline stratification. For this data set, correction for variations due to tide, variations in the sea level due to bad weather condition and variations due to other acquisition related factors were applied to the data set.

This paper presents a modern approach and easy recipe for correcting water column height variation using co-recorded information from dGPS especially in near shore, shallow and highly erosive marine shelf settings. The methodology was applied to the SES data acquired from the shallow-water area of the German North Sea where tidal influence and water column height changes has been documented. Variation in water column heights along profile length reached over 3.0 m with large corrections attributed to rapid shoaling occurring near the coast and interplay between the water column and sea floor morphology. The corrected profiles which are now referenced to a consistent MSL show improved quality when compared to the uncorrected profiles. The sea floor from the corrected profile occurred deeper and showed good consistency with the bathymetry data compared to the uncorrected profiles. It was possible to distinguish and classify different generations of valley/channel structures which are on apparently similar depth levels stratigraphically based on their small gradient using the corrected profiles and thus validating the applicability of this methodology. The correction and datum referencing also helped in developing the relative true stratigraphic model for the area investigated. The findings and methodology are especially important for further high-resolution sea level variation studies and can be used to directly compute water column statics required for improve quality interpretation derived from seismic data. The less time-consuming methodology can be applied to all sorts of reflections seismic data and requires only a small budget which is especially important for scientific data acquisition and processing.
